# Household Hints and Recipes

**Published:** 1881-10

**Authors:** 


### Household Hints & Recipes

TO REMOVE OLD INK-SPOTS.—Pyrophosphate of soda is recommended, when the stains are on colored fabrics, as the salt does not injure their color.

FIRE-PROOF CEMENT.—Silicate of soda mixed with asbestos is the nearest to a fire-proof cement. It will stand a low, red heat. It is decomposed at a bright red.

CHARCOAL TOOTH POWDER.—

Charcoal, 3 ounces.
Calamus root, 5 drachms.
Pumice stone, 2½ "
Catechu, 2½ "
Oil bergamot, 30 drops.
Oil Cloves, 30 drops.

OXIDIZING SILVER.—Dr. Ellsner says that there are two distinct shades in use, one produced by chloride, which has a brownish tint, and the other by sulphur, which has a bluish-black tint. To produce the former it is only necessary to wash the article with a solution of sal ammoniac; a much more beautiful tint may, however, be obtained by employing a solution composed of equal parts of sulphate of copper and sal-ammoniac in vinegar. The fine black tint may be produced by a slightly warm solution of sulphuret of potassium or sodium.

LADIES' SHOE DRESSING.—The following was originally communicated by a correspondent of *The Druggists' Circular*:

Extract of logwood, 2 ounces.
Bichromate of potassa, 2 drachms.
Yellow prussiate of potassa, 2 drachms.
Powdered borax, 3 ounces.
Water of amonia, 2 ounces.
Shellac, 16 ounces.
Water, 1 gallon.

Dissolve the extract in the water, heating the liquid to nearly the boiling point. Then add the chromate and the prussiate of potassa. After a deep rich blue color has developed, add the borax, and when it has disolved, the shellac, and lastly, the ammonla. Then keep the whole at a gentle heat, agitating the mixture with a stick of wood, until the smell of ammonia has disappeared and the shellac has dissolved.

RAPID GERMINATION OF SEEDS.—When steeped in water containing two per cent. of ammonia, seeds are said to germinate within twenty-four hours.

TO PRESERVE STARCH PASTE.—By the addition of eight or ten per cent. of alum or borax, the starch can be kept for quite a length of time without spoiling.

VARNISH FOR PREVENTING RUST.—A Varnish for this purpose may be made of 120 parts resin, 280 sandarac, 50 gum lac. They should be heated gradually until melted, and thoroughly mixed, then 120 parts turpentine added, and subsequently after heating, 180 parts rectified alcohol. After careful filtration, it should be put into tightly-corked bottles.

DANGERS OF MOULDY BREAD.—A singular case of poisoning from eating a pudding made in part of mouldy bread, is reported in the *Sanitary Record.* The main facts of the case may be briefly stated as follows: The principle materials of the pudding consisted of scraps of bread left from making toast and sandwiches, and they had been about three weeks accumulating. To these scraps were added milk, eggs, sugar, currants, and nutmeg. The whole was baked in a slow oven, and was subsequently eaten by the cook, the proprietor of the eating-house in which it was prepared, the children of the proprietor, and two other persons. All of these became violently ill, with symptoms of irritant poisoning. One of the children (aged three years) and one of the adults died. The necropsy of the body of the child caused the medical men to suspect poisoning, and accordingly the viscera, together with the remnant of the pudding, the materials used in making it, the matter vomited, etc., were sent to a chemical analyst, Mr. Alfred Allen, for examination. He made tests for several poisons, but without positive result. A puppy was fed with the pudding for two days without any poisonous effect. He was then led to look for ergot in the pudding, and was soon startled to find unquestionable evidence of its presence, as far as the chemical reactions went, though he was unable, with the aid of the microscope, to detect any actual ergot. From the facts Mr. Allen infers that the reactions hitherto supposed to be peculiar to ergot are common to other poisonous fungi.—*Popular Science Monthly*,

TO RENDER LEATHER WATERPROOF.—1. Melt together 2 oz. Burgundy pitch, 2 oz. beeswax, 2 oz. of turpentine, and 1 pint of raw linseed oil. Lay on with a brush while warm.

2. Melt three ounces of lard and add one ounce of powdered resin. This mixture remains soft at ordinary temperatures, and is an excellent application for leather.

---

LIQUID STARCH GLOSS.—

Spermaceti, 2 ounces.
Gum senegal, 2 ounces.
Borax, 2 ounces.
Glycerine, 5 ounces.
Water, 49 ounces.

Mix and boil together. Two or three teaspoonfuls to be added to a quarter pound of boiled starch.

---

LIQUID GLUE.—This article, so common and usefnl, can be made easily by diluting officinal phosphoric acid with two parts, by weight, of water, and saturating with carbonate of ammonia; dilute the resulting liquid, which must be still somewhat acid, with another part of distilled water, warm it on a water-bath, and dissolve in it enough good glue to form a thick, syrupy liquid. It must be kept in well closed bottles.

---

A NEW DYE FOR FEATHERS.—At a Berlin feather-dyeing establishment an ostrich feather dyed in shades with methyl-violet was laid upon a paper upon which some ammonia had been poured, but had dried up again. After a time the feather became partially green, the green passing gradually into violet, and producing an extraordinary effect. This reaction is being utilized in feather-dying, and will probably be applied in the manufacture of artificial flowers.

---

COFFEE AND EGG FOR SICK PERSONS.—A sick person wanting nourishment and having lost appetite, can often be sustained by the following, when nothing else could be taken: Make a strong cup of coffee, adding boiling milk as usual, only sweetening rather more; take an egg, beat yolk and white together thoroughly; boil the coffee, milk and sugar together, and pour it over the beaten egg in the cup you are going to serve it in. This simple receipt is used frequently in hospital practice.

THE TREATMENT OF TOOTHACHE.—*Cassel's Family Physician*, an English publication, contains an article on toothache from which the following items are taken:

A few drops of chloroform on cotton wool inserted into the hollow of a decayed aching tooth often gives permanent relief, but sometimes when the anæsthetic effect has passed away the pain is aggravated, the application having irritated the inflamed pulp. A better plan is to hold over the hollow tooth a piece of lint moistened with chloroform, so that the vapor only comes in contact with the interior of the tooth. The preparation sold as camphorated chloroform often proves useful. A mixture of equal parts of chloroform and laudanum, or of chloroform and creasote, constitutes an excellent application.

Creasote may nearly always be employed with a fair hope of success. It may be mixed with an equal quantity of chloroform, or with tannin. Laudanum, either alone or mixed with tannin or creasote, and inserted into the cavity of the hollow tooth, enjoys a high and well merited reputation.

For cases in which the pulp is exposed and inflamed, a jelly is made by melting in a test tube some crystallized carbolic acid, and then adding an equal quantity of collodion. A small quantity is placed on cotton wool and inserted into the hollow painful tooth. It may at first somewhat aggravate the pain, but in a few seconds it diminishes and soon abolishes it. Care should be taken not to let it come in contact with the inside of the cheek, for as we can testify from personal experience, it would give rise to considerable pain and smarting.

When there is a large hollow and the pain is severe, a good application is a mixture of camphor and opium, of each one grain, made into a paste, with which the cavity should be filled, it having been previously dried by means of lint or cotton wool.

When equal parts of chloral or powdered camphor are rubbed up together, they form a syrupy liquid. This will sometimes succeed in relieving toothache even when applied externally; but it is more likely to afford relief when introduced into the cavity of the decayed tooth on cotton wool.

A plug of lint dipped in sulphurous acid and inserted in the hollow tooth, will often give immediate relief.

